# Asna1/TRC40 that mediates membrane insertion of tail-anchored proteins is required for efficient release of Herpes simplex virus 1 virions

**DOI:** 10.1186/s12985-016-0638-8

**Published:** 2016-10-20

**Authors:** Melanie Ott, Débora Marques, Christina Funk, Susanne M. Bailer

**Affiliations:** 1 Max von Pettenkofer-Institut, Ludwig-Maximilians-Universität München, Pettenkoferstr. 9a, 80336 München, Germany; 2 Institute for Interfacial Engineering and Plasma Technology IGVP, University of Stuttgart, Nobelstrasse 12, 70569 Stuttgart, Germany

**Keywords:** Herpesvirus, HSV1, Integral membrane proteins, Tail-anchored protein biogenesis, Asna1/TRC40, pUL34, pUL56, pUS9, Nuclear egress, Exocytosis

## Abstract

**Background:**

Herpes simplex virus type 1 (HSV1), a member of the alphaherpesvirinae, can cause recurrent facial lesions and encephalitis. Two membrane envelopment processes, one at the inner nuclear membrane and a second at cytoplasmic membranes are crucial for a productive viral infection. Depending on the subfamily, herpesviruses encode more than 11 different transmembrane proteins including members of the tail-anchored protein family. HSV1 encodes three tail-anchored proteins pUL34, pUL56 and pUS9 characterized by a single hydrophobic region positioned at their C-terminal end that needs to be released from the ribosome prior to posttranslational membrane insertion. Asna1/TRC40 is an ATPase that targets tail-anchored proteins to the endoplasmic reticulum in a receptor-dependent manner. Cell biological data point to a critical and general role of Asna1/TRC40 in tail-anchored protein biogenesis. With this study, we aimed to determine the importance of the tail-anchored insertion machinery for HSV1 infection.

**Methods:**

To determine protein-protein interactions, the yeast-two hybrid system was applied. Asna1/TRC40 was depleted using RNA interference. Transient transfection and virus infection experiments followed by indirect immunofluorescence analysis were applied to analyse the localization of viral proteins as well as the impact of Asna1/TRC40 depletion on virus infection.

**Results:**

All HSV1 tail-anchored proteins specifically bound to Asna1/TRC40 but independently localized to their target membranes. While non-essential for cell viability, Asna1/TRC40 is required for efficient HSV1 replication. We show that early events of the replication cycle like virion entry and overall viral gene expression were unaffected by depletion of Asna1/TRC40. Furthermore, equal amounts of infectious virions were formed and remained cell-associated. This indicated that both nuclear egress of capsids that requires the essential tail-anchored protein pUL34, and secondary envelopment to form infectious virions were successfully completed. Despite large part of the virus life cycle proceeding normally, viral propagation was more than 10 fold reduced. We show that depletion of Asna1/TRC40 specifically affected a step late in infection during release of infectious virions to the extracellular milieu.

**Conclusions:**

Asna1/TRC40 is required at a late step of herpesviral infection for efficient release of mature virions to the extracellular milieu. This study reveals novel tools to decipher exocytosis of newly formed virions as well as hitherto unknown cellular targets for antiviral therapy.

## Background

Herpesviruses have evolved a life cycle that strongly depends on two membrane-envelopment processes, one at the inner nuclear membrane (INM) called primary envelopment, and another at cytoplasmic membranes called secondary envelopment, both of which are crucial for a productive viral infection [1, 2]. Depending on the subfamily, herpesviruses encode more than 11 different transmembrane proteins involved in various aspects of the individual viral life cycle.

Tail-anchored (TA) proteins represent a specific class of transmembrane proteins characterized by a single transmembrane domain (TMD) positioned at its very C-terminal end. Thus, the hydrophobic region of a TA protein remains associated with the ribosomal tunnel until translation is complete [3–6]. This requires that TA proteins are released from the ribosome prior to their post-translational insertion into various target membranes. The identification of the TMD recognition complex of 40 kDa (TRC40) also known as Asna1 provided a major breakthrough in understanding the TA protein biogenesis. Asna1/TRC40 is an ATPase conserved in many species. It captures a TA protein following its ribosomal translation and together with several other components delivers it to a receptor of the endoplasmic reticulum (ER). Recent biochemical and structural analysis have further elucidated the mechanism of membrane insertion of TA proteins. The ATP-bound dimer of Asna1/TRC40 or its orthologs form a hydrophobic groove that accommodates the TMD of TA proteins. The resulting Asna1/TRC40-TA protein complex is then recruited to the ER receptor resulting in release of the TA protein and membrane insertion, a process that may require ATP hydrolysis.

**Fig. 1:**
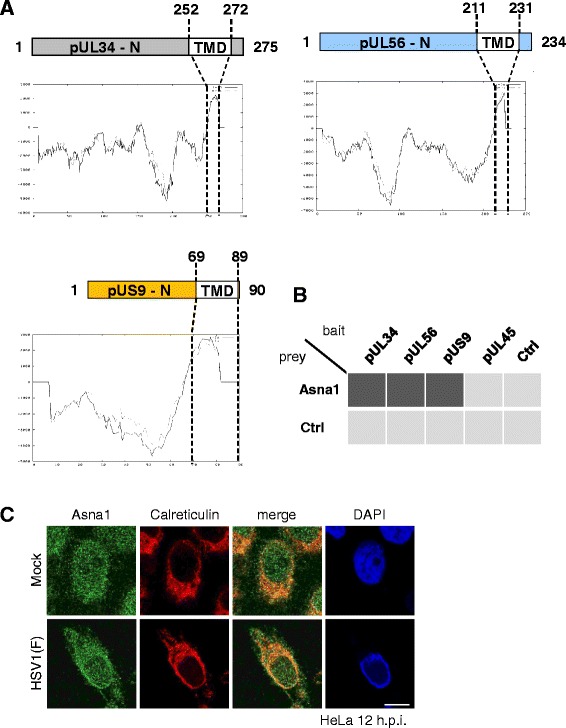
HSV1 encodes three tail-anchored proteins that interact with Asna1/TRC40. **a** Schematic diagrams show the domain organization of pUL34, pUL56 and pUS9. The transmembrane domain (TMD) of pUL34 (residues 252–272), pUL56 (residues 211–231) and pUS9 (residues 69–89) and the hydrophobicity plots generated by TMpred (http://embnet.vital-it.ch/software/TMPRED_form.html) are depicted. **b** The yeast two-hybrid (Y2H) system was used to analyse the interaction of Asna1 and the HSV1 encoded tail-anchored (TA) proteins pUL34, pUL56 and pUS9. pUL45, a type II membrane protein with an N-terminal transmembrane protein was used as control. Asna1 fused to the Gal4 activation domain (AD) was tested for interaction with pUL34, pUL56, pUS9 and pUL45 fused to the Gal4 DNA-binding domain (DBD). Interaction of proteins is indicated by transcriptional activation of the *HIS3* reporter gene enabling growth (*black squares*) or no growth (*grey squares*) of yeast cells on selective media. **c** To determine the subcellular distribution of Asna1/TRC40 during HSV1 infection, HeLa cells were mock treated or infected with HSV1(F) at an MOI of 1 for 12 h followed by IF analysis using Asna1/TRC40- and Calreticulin-specific antibodies followed by secondary reagents. Nuclei were visualized by DAPI staining

Like all TA proteins, the HSV1 TA proteins pUL34, pUL56 and pUS9 are characterized by a cytoplasmic domain, a single C-terminal transmembrane domain (TMD) and a short luminal extension (Fig. 1a). HSV1 pUL34 is a protein conserved throughout the herpesvirus family ([7]; and references therein). Both its cyto-/nucleoplasmically exposed N-terminal domain (residues 1–252) and its C-terminal TMD (residues 252–272) are essential for viral replication [8–10]. Posttranslational membrane insertion of pUL34 occurs in the cytoplasm and thus prior to its targeting to the INM [7]. There, pUL34 associates with the nucleocapsid-bound pUL31 for subsequent primary envelopment and egress of capsids to the cytoplasm [7].

The other two HSV1 TA proteins, pUL56 and pUS9, are non-essential and specific for alpha-herpesviruses ([11, 12]; and references therein). pUL56 is composed of a cytoplasmic domain (residues 1–211) followed by a hydrophobic region (residues 211–231) and a short luminal domain (Fig. 1a). In pUS9, a short N-terminal domain (residues 1–69) is followed by a hydrophobic domain between residues 69–89. Both pUL56 and pUS9 localize to the *trans* Golgi network (TGN) and are integrated into mature virions during secondary envelopment [13].

Many herpesviral functions have been analysed in great detail while our knowledge of virus-host interactions and their importance for viral replication is far from complete. With this study we focus on the biogenesis of tail-anchored (TA) proteins and its importance for herpesviral infection. Upon knockdown of Asna1/TRC40, large part of the viral infection cycle proceeds normally and infectious virions are formed, their release to the extracellular milieu late in infection however is delayed. Together our data suggest that efficient transport of infectious virions along the secretory pathway requires Asna1 and thus the TA insertion machinery.

## Methods

### Cells, yeast 2-hybrid assay and general cloning

HeLa (ATCC CCL-2) and Vero cells (ATCC CRL-1587) were grown in DMEM containing 10 % FCS. Yeast 2-hybrid (Y2H) analysis was done as described [14]. The UL34, UL45, UL56 and US9 genes previously cloned into the entry vector pDONR207 [15] were transferred into the Gateway compatible Y2H bait vector pGBKT7-DBD and/or the mammalian expression vector pCR3-N-myc according to the manufacturer’s protocol (Invitrogen). The human Asna1/TRC40 gene previously cloned into the pDONR223 vector was transferred to the Gateway compatible Y2H prey vector pGADT7-AD according to the manufacturer’s protocol (Invitrogen).

### Viruses

HSV1(F) (provided by B. Roizman, University of Chicago, USA) was used for infection experiments. The strain HSV1(17+)lox (provided by B. Sodeik, Hannover Medical School, Germany) was used as PCR template. HSV1 propagation and virus growth curves were performed as described [14]. To monitor infection, Vero cells were infected with HSV1(F) at the indicated MOI. Cell lysates were prepared at the indicated times post infection and analysed by Western blotting using primary antibodies to the immediate early proteins ICP0 (anti-ICP0, Santa Cruz) and ICP27 (anti-ICP27, Virusys), to the early protein gB (anti-Glykoprotein B, Santa Cruz) and to the late proteins VP5 (anti-ICP5 (VP5), Abcam) and pUL34 [9] followed by secondary antibodies conjugated to POX. Antibodies specific to β-actin (Abcam) were used as control.

### Indirect immunofluorescence microscopy

Indirect immunofluorescence (IF) analysis of transfected or infected cells was done as described [14]. For plasmid transfection, the Effectene Transfection Reagent was used. For virus infection, HeLa cells were infected at the indicated MOI. In infected cells, binding of antibodies to the HSV1 Fc-receptor like proteins gE/gI was blocked overnight at 4 °C with human IgG (200 μg/ml) and 10 % FCS in PBS [16]. The mouse monoclonal antibodies anti-myc [9E10] (Santa Cruz), anti-ICP8 (provided by R. Heilbronn, Charité Universitätsmedizin Berlin CCM, Berlin, Germany), anti-Asna1/TRC40 ([M03], Klon 2H3 Abnova) and rabbit polyclonal antibodies anti-pUL34 [9], anti-Calreticulin (Sigma), and anti-Giantin (Abcam) were used as primary reagents. Goat anti-rabbit or anti-mouse antibodies coupled to Alexa488 or Alexa594 (all Invitrogen) were used as secondary reagents. Cells were examined using a Leica confocal laser scanning microscopes TCS SP5 and LSM710. Images were recorded using the Leica Application Suite AF6000 Software and processed using Adobe Photoshop.

### SiRNA transfection

Gene silencing was essentially done as described [17]. Briefly, siRNAs (20 nM; GE Dharmacon), 150 μl of HBSS, and 1,5 μl of transfection reagent were mixed and added to HeLa cells in DMEM with 5 % FCS seeded in 12-well plates. Efficiency of RNAi was monitored by Western blotting using mouse monoclonal antibodies to Asna1/TRC40 ([M03], Klon 2H3 Abnova) and polyclonal goat anti-Lamin B (Santa Cruz) to control for loading. The siRNA duplexes used for Asna1/TRC40 knockdown and as controls (Ctrl) are shown in Table 1. Viral infection was generally performed 48 h (h) after siRNA treatment. Infectious virions were quantified by removing aliquots of medium and of infected cells at various time points followed by plaque assay on Vero cells [14]. To determine genome copy/pfu ratios of virions released from cells, HeLa cells were treated with Asna1/TRC40 specific or ctrl siRNA followed by infection with HSV1(F) for 30 h. Realtime quantitative PCR using HSV1 specific primers were used to determine the genome copies, plaque assays were performed as described [14].

**Table 1:** SiRNAs used for gene silencing

Name	Sequence (5′→3′)
Asna1_1	GAUCAGCCCUUUCAUCUCA
Asna1_2	GCGUAUGCAUUGCUGAGUU
Asna1_3	GAACUUCUCGGUGGUGGUA
Asna1_4	CAGGAGGCCAUGAGCGCAU
Ctrl	UUCUCCGAACGUGUCACGU

## Results

### HSV1 encodes three tail-anchored proteins that interact with Asna1/TRC40

HSV1 encodes three TA proteins, called pUL34, pUL56 and pUS9. Hydrophobicity plots show that pUL34, an essential protein conserved throughout the herpesviral family, contains a transmembrane domain (TMD) between residues 252–272 required for nuclear egress (Fig. 1a; [9]). Two other TA proteins, pUL56 and pUS9, that are non-essential and specific for alpha-herpesviruses, carry a TMD between residues 211–231 and 69–89, respectively (Fig. 1a).

To determine whether pUL34, pUL56 and pUS9 interact with Asna1/TRC40, the yeast 2-hybrid (Y2H) system was applied. Asna1/TRC40 fused to the Gal4 activation domain (AD) was tested for interaction with pUL34, pUL56 and pUS9 fused to the Gal4 DNA-binding domain (DBD). pUL45, that carries an N-terminal TMD co-translationally integrated into membranes by an Asna1/TRC40-independent mechanism, was used as control. Interaction of proteins was reported by growth of yeast cells on selective media. While DBD-pUL34, -pUL56 and -pUS9 co-expressed with AD-Asna1/TRC40 allowed for growth of yeast cells, this was not the case for co-expression of DBD-pUL45 and AD-Asna1/TRC40 (Fig. 1b). We thus conclude that all three TA proteins of HSV1 specifically interacted with Asna1/TRC40 supporting its function in posttranslational membrane insertion of these viral proteins.

To analyse the subcellular distribution of Asna1/TRC40 in presence and absence of HSV1 infection, HeLa cells were mock treated or infected with HSV1(F) for 12 h and subsequently processed for IF. Infected cells were readily identified based on their marginalized chromatin as revealed by DAPI staining (Fig. 1c). Both in noninfected and infected cells, Asna1/TRC40 showed a pancellular distribution and significantly co-localized with the ER marker Calreticulin (Fig. 1c) suggesting that its distribution is essentially unaltered during HSV1 infection (Fig. 1c).

### Targeting and membrane insertion of HSV1 pUL34, pUL56 and pUS9 occur independent of Asna1/TRC40

**Fig. 2:**
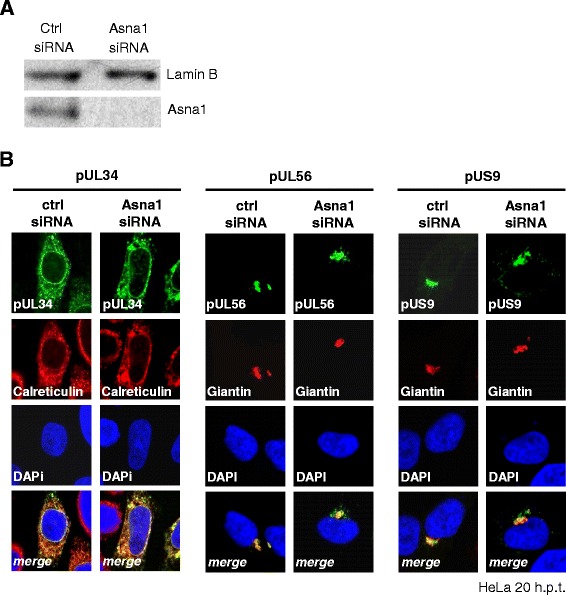
Targeting and membrane insertion of HSV1 pUL34, pUL56 and pUS9 occur independent of Asna1/TRC40. **a** To determine the effect of Asna1/TRC40 depletion on cell growth, HeLa cells were transfected with Asna1/TRC40 specific or control (ctrl) siRNA for 48 h. Gene knockdown was monitored by Western blotting using Asna1/TRC40 and Lamin B specific antibodies followed by secondary reagents. While efficient knock-down was achieved, cell viability was normal indicating that Asna1/TRC40 silencing did not affect cellular growth. **b** To determine whether the HSV1 TA proteins require the Asna1/TRC40 mediated pathway for localization to their target membrane, Asna1/TRC40 knockdown was performed in HeLa cells. Subsequently, cells were transfected with plasmids encoding myc-tagged TA proteins and 20 h later analysed by IF using monoclonal anti-myc and polyclonal anti-Calreticulin or anti-Giantin antibodies followed by secondary reagents. Nuclei were visualized by DAPI staining

In absence of other viral proteins, pUL34 is targeted to the ER and the nuclear periphery, whereas pUL56 and pUS9 are located to the *trans* Golgi network (TGN). To determine whether Asna1/TRC40 is required for proper membrane targeting of the HSV1 TA proteins, HeLa cells were transfected for 48 h with Asna1/TRC40 specific or control (ctrl) siRNA. Knockdown of Asna1/TRC40 was highly efficient as shown by Western blotting (Fig. 2a). Interestingly, depletion of Asna1/TRC40 did not affect cell viability of Hela cells (data not shown).

RNAi treated cells were then transfected with plasmids encoding myc-tagged TA proteins and 20 h later analysed by IF using monoclonal anti-myc antibodies. Calreticulin or Giantin were used as markers of the ER and the TGN, respectively. pUL34 showed a reticular subcellular distribution and co-localized with the ER marker Calreticulin consistent with its localization to the ER and the nuclear periphery whether the cells were treated with Asna1/TRC40 specific or ctrl siRNA (Fig. 2b, left panel). pUL56 and pUS9 both located to the TGN as indicated by their co-localization with the TGN marker Giantin (Fig. 2b, middle and right panel). In Asna1 depleted cells, a certain amount of pUS9 was found in a perinuclear region suggesting that membrane insertion of pUS9 is influenced by the absence of Asna1. To summarize, all HSV1 TA proteins seemed to efficiently reach their target membranes irrespective of the presence or absence of Asna1/TRC40.

### Asna1/TRC40 is dispensable for virion entry and gene expression during HSV1 infection

**Fig. 3:**
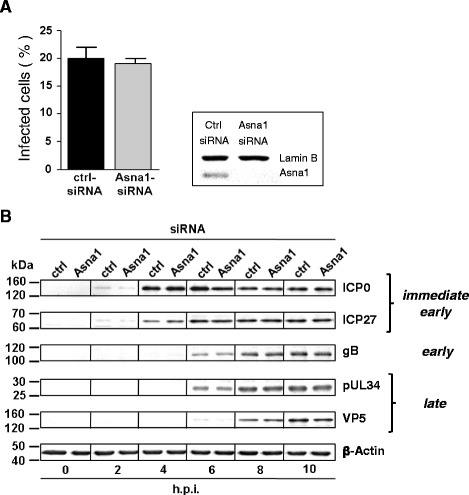
Asna1/TRC40 is dispensable for virion entry and gene expression during HSV1 infection. **a** To determine the importance of Asna1/TRC40 for entry and early gene expression of HSV1, HeLa cells were transfected with Asna1/TRC40 specific or control (ctrl) siRNA for 48 h. Silencing was monitored as described in Fig. 2a (inset). Subsequently, cells were infected with HSV1(F) at an MOI of 0.5 for 4 h and analysed by IF using monoclonal anti-ICP0 antibody followed by secondary reagents. Nuclei were visualized by DAPI staining. A total of 700 cells of either cell type was analysed. **b** To determine whether Asna1/TRC40 is required for overall viral gene expression, cells treated with siRNA for 48 h were subsequently infected with HSV1 at an MOI of 5, harvested at the indicated times post infection and analysed by Western blotting using ICP0, ICP27, glycoprotein B (gB), pUL34 and VP5 (ICP5), and specific antibodies followed by secondary reagents. For control, β-actin specific antibodies were used

To determine whether Asna1/TRC40 is required for the herpesviral life cycle, Asna1/TRC40 knockdown was performed and monitored as shown before (Fig. 2a). Then, siRNA treated HeLa cells were infected with HSV1 at an MOI of 0.5 for 4 h (Fig. 3a). ICP0 expression was analysed as indirect means of viral entry. IF analysis revealed that 20 % and 19 % of the cells treated with ctrl- and Asna1/TRC40-specific siRNAs, respectively, were infected by HSV1(F).

A time course experiment to detect viral proteins of all kinetic classes was performed. HeLa cells were first treated with Asna1/TRC40 or ctrl siRNA for 48 h and subsequently infected with HSV1 at an MOI of 1. Cell lysates were prepared at the indicated time-points and probed with antibodies specific to the immediate early regulators ICP0 and ICP27, to glycoprotein gB, to the nuclear egress protein pUL34 and the major capsid protein ICP5 (VP5). β-Actin-specific antibodies were used to control for equal loading of cell samples (Fig. 3b). The transcriptional regulators ICP0 and ICP27 were detected 2 h post infection (h.p.i.), glycoprotein gB and the nuclear egress protein pUL34 appeared 6 h.p.i., while the major capsid protein ICP5 was detected 8 h.p.i.. Taken together, we found that Asna1/TRC40 is not required for virion entry and overall viral gene expression (Fig. 3b).

### Targeting of pUL34 to the nuclear envelope during infection is independent of Asna1/TRC40

**Fig. 4:**
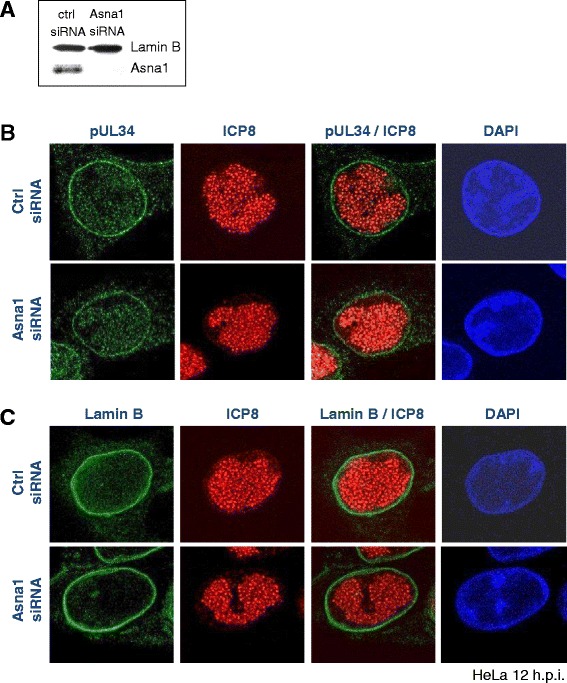
Targeting of pUL34 to the nuclear envelope during infection is independent of Asna1/TRC40. To determine the importance of Asna1/TRC40 for localization of pUL34 to the nuclear envelope in the virus context, HeLa cells were transfected with Asna1/TRC40 specific and control (ctrl) siRNA for 48 h (**a**). HSV1(F) infection was then performed for 12 h at an MOI of 0.1 followed by IF analysis using rabbit anti-pUL34 antibodies (**b**) or goat anti-Lamin B antibodies (**c**) in combination with mouse anti-ICP8 antibodies and secondary reagents. Nuclei were visualized by DAPI staining

HSV1 pUL34 is essential with a conserved function in nuclear egress of capsids. To determine whether Asna1/TRC40 is required for pUL34 biogenesis in the viral context, RNAi was performed as described (Figs. 2a and 4a). Subsequently, HeLa cells were infected with HSV1 at an MOI of 1 for 12 h and subsequently processed for IF (Fig. 4b and c). Antibodies specific to pUL34 (Fig. 4b) and Lamin B (Fig. 4c) showed that both proteins were exclusively located to the nuclear envelope. Furthermore, intranuclear replication centers formed normally, as revealed by ICP8-specific antibodies (Fig. 4b and c). Thus, we conclude that pUL34 membrane insertion and targeting to the INM, a prerequisite for NEC formation and capsid nuclear egress, proceeds normally in absence of Asna1/TRC40.

### Asna1/TRC40 is required late in infection for efficient release of infectious virions from the cell

**Fig. 5:**
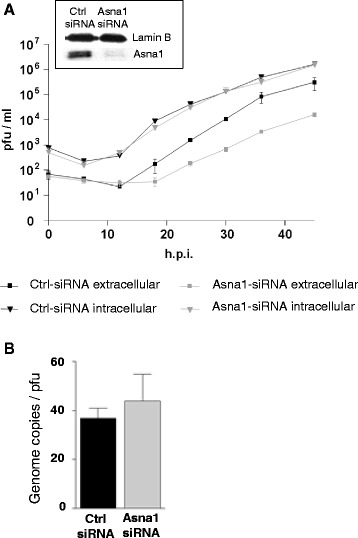
Asna1/TRC40 is required late in infection for efficient release of infectious virions from the cell. **a** To analyse the growth properties of HSV1 on Asna1/TRC40 depleted cells, HeLa cells were first transfected with Asna1/TRC40 specific or control (ctrl) siRNA for 48 h. Silencing was monitored as described in Fig. 2a (inset Fig. 5a). Subsequently, HeLa cells were infected with HSV1 at an MOI of 0.1. Cells and cell supernatants were harvested at the indicated time points. Virions present in cells or in the extracellular milieu were titrated by plaque assays on Vero cells in triplicates. **b** Genome copy/pfu ratio was determined for virions released from siRNA treated cells at 30 h.p.i. using plaque assays and realtime quantitative PCR

To determine the overall effect of Asna1/TRC40 knockdown on the outcome of an HSV1 infection, Asna1/TRC40 depleted HeLa cells or ctrl cells were infected with HSV1 at an MOI of 0.1. At the indicated time points, medium and infected cells were harvested separately and analysed for the presence of infectious virions using plaque assays. Equal amounts of infectious virions were formed and remained cell-associated whether cells were treated with Asna1/TRC40 specific or ctrl siRNA (Fig. 5a). In contrast, about 10 times less infectious virions were released to the culture medium upon Asna1/TRC40 depletion. To determine the genome copy/pfu ratio of virus released 30 h.p.i. from the infected cells, realtime quantitative PCR was performed. Virions released from Asna1 siRNA treated cells showed reduced amounts of genomes as well as plaque forming units compared to the ctrl treated cells. In either case however, their genome copy/pfu ratio was comparable indicating that virions released to the extracellular milieu were similar in infectivity (Fig. 5b). Thus, although not essential for cellular growth and formation of infectious virions, Asna1/TRC40 is required for release of mature virions from the cells at a late step of virus infection and thus for efficient HSV1 propagation.

## Discussion

Asna1/TRC40 plays a central role during TA protein biogenesis by binding to TMDs of newly synthesized TA proteins and targeting them to the ER receptor for subsequent membrane insertion [3–6]. While most data on Asna1/TRC40-mediated TA protein biogenesis are based on in vitro data, we show here that in vivo, a highly efficient knockdown of Asna1/TRC40 does not interfere with cell growth. Thus, Asna1/TRC40 seems to be non-essential for cell viability under normal conditions. This is surprising considering the many functionally important TA host proteins [18], but consistent with the finding that components of the TA insertion machinery are non-essential in yeast cells unless additional stress is present [5]. Pathways redundant with Asna1/TRC40 for posttranslational membrane insertion may involve the signal recognition particle SRP or the heat shock protein dimers Hsc70/Hsp40 [19–21].

Despite Asna1/TRC40 being non-essential for cellular life, our data show that it is important for efficient herpesviral propagation. Large part of the herpesviral life cycle including nuclear egress of capsids tolerates the absence of Asna1/TRC40; viral morphogenesis is comparable to control treated cells giving rise to equal numbers of intracellular infectious virions. Interestingly, however, a specific defect is observed at a very late step of the viral life cycle during release of mature virions to the extracellular milieu. Thus, our data reveal a novel role of Asna1/TRC40 late in HSV1 infection required for efficient cytoplasmic transport and/or release of infectious virions.

Recent evidence supports a role of the constitutive secretory pathway in delivering virus containing secretory vesicles to sites of exocytosis at the plasmamembrane [22]. A role for Asna1/TRC40 orthologs in transport and release/fusion of secretory vesicles is supported by studies in yeast [23] and *Caenorhabditis elegans* (*C. elegans*) [24]. In the herpesviral context, Asna1/TRC40 depletion could limit the amount of specific host TA proteins required at the site of virion exocytosis. In this respect, the SNARE proteins involved in various vesicular and membrane fusion processes represent an important group of TA host factors [18, 25, 26]. Indeed, depletion of the SNARE TA protein Syntaxin 3 results in reduced release of infectious Human cytomegalovirus (HCMV) virions [27]. Alternatively, the highly productive virus infection may pose a general stress on the TA insertion machinery thereby overwhelming its capacity. We were unable to identify a specific compartment where the virions accumulated in absence of Asna1 (data not shown) suggesting that the overall dynamic of virion release is hampered.

Our data show that all HSV1 encoded TA proteins specifically bind to Asna1/TRC40 consistent with their TMD hydrophobicity index ≥ 40 [4]. Despite its ability to bind pUL34, Asna1/TRC40 is redundant for pUL34 localization and function. Both pUL56 and pUS9 effectively reached their target membranes in absence of Asna1/TRC40. However, a certain amount of pUS9 was mislocalized upon isolated expression suggesting that Asna1 modulates pUS9 membrane insertion. Since individual TA proteins favor particular insertion factors [5], it is quite possible that pUL56 and pUS9 differ from pUL34 in their propensity to use Asna1/TRC40 for membrane targeting. Unfortunately, detailed analysis of pUL56 and pUS9 in the viral context is limited due to the lack of specific antibodies.

How could a reduced membrane insertion of the viral TA proteins pUL56 and pUS9 affect virion release to the extracellular milieu? Exposed on the cytoplasmic face of secretory vesicles, their N-terminal domains may interact with kinesin motor proteins [12, 28] to shuttle the vesicular content to sites of secondary envelopment. This way, pUL56 and pUS9 could modulate the transport of secretory vesicles containing infectious virions. Interestingly, pUS9 was recently reported to be necessary for anterograde transport of virions in neurons ([29]; and references therein). Thus, TA protein biogenesis may have a particular impact on herpesviral neuropathology where long-distance axonal transport of virus containing secretory vesicles is likely to occur.

Biogenesis of TA membrane proteins destined for the INM is not well understood. Membrane insertion of the viral TA protein pUL34 that occurs prior to its transport to the INM [7] is essential for its function ([7, 9]; and references therein). Thus, pUL34 can serve as a viral reporter to gain insight into the biogenesis of TA proteins associated with the INM. Asna1/TRC40 specifically binds to pUL34 suggesting it supports membrane insertion of the INM protein pUL34. A role of Asna1/TRC40 in INM biogenesis is also provided by data on Emerin, a TA protein associated with Emery-Dreifuss muscular dystrophy [30]. Together these data indicate that biogenesis of INM TA proteins engages Asna1/TRC40-dependent and -independent pathways [9, 30].

Taken together, our data reveal a role of the TA protein biogenesis during release of virions. Asna1/TRC40 depletion may provide a tool to study this poorly characterized process, a decisive step for virus spread. Since Asna1/TRC40 knockdown preferentially perturbs virus replication while cellular growth remains unaffected, analysis of TA protein biogenesis may reveal antiviral targets to inhibit virus propagation.

## Conclusions

- ■ The TA protein insertion factor Asna1/TRC40 is nonessential.
- ■ HSV1 encodes three tail-anchored proteins pUL34, pUL56 and pUS9.
- ■ All HSV1 TA proteins specifically bind to Asna1/TRC40.
- ■ Asna1/TRC40 is required for efficient HSV1 replication.
- ■ Asna1/TRC40 is redundant for nuclear egress of capsids.
- ■ Depletion of Asna1/TRC40 results in a defect late in herpesviral infection during release of infectious virions.

## Abbreviations

AD: Activation domain
Ctrl: Control
DBD: DNA binding domain
ER: Endoplasmic reticulum
h: Hours
h.p.i.: Hours post infection
HSV1: Herpes simplex virus type 1
IF: Indirect immunofluorescence
INM: Inner nuclear membrane
NEC: Nuclear egress complex
RNAi: RNA interference
TA: Tail-anchor
TGN: *trans* Golgi network
TMD: Transmembrane domain
TRC40: TMD recognition complex of 40 kDa
Y2H: Yeast 2-hybrid
